# Deep learning applications to breast cancer detection by magnetic resonance imaging: a literature review

**DOI:** 10.1186/s13058-023-01687-4

**Published:** 2023-07-24

**Authors:** Richard Adam, Kevin Dell’Aquila, Laura Hodges, Takouhie Maldjian, Tim Q. Duong

**Affiliations:** Department of Radiology, Albert Einstein College of Medicine and the Montefiore Medical Center, 1300 Morris Park Avenue, Bronx, NY 10461 USA

**Keywords:** Machine learning, Artificial intelligence, Texture feature analysis, Convolutional neural network, MRI, Dynamic contrast enhancement

## Abstract

Deep learning analysis of radiological images has the potential to improve diagnostic accuracy of breast cancer, ultimately leading to better patient outcomes. This paper systematically reviewed the current literature on deep learning detection of breast cancer based on magnetic resonance imaging (MRI). The literature search was performed from 2015 to Dec 31, 2022, using Pubmed. Other database included Semantic Scholar, ACM Digital Library, Google search, Google Scholar, and pre-print depositories (such as Research Square). Articles that were not deep learning (such as texture analysis) were excluded. PRISMA guidelines for reporting were used. We analyzed different deep learning algorithms, methods of analysis, experimental design, MRI image types, types of ground truths, sample sizes, numbers of benign and malignant lesions, and performance in the literature. We discussed lessons learned, challenges to broad deployment in clinical practice and suggested future research directions.

## Background

Breast cancer is the most common cancer and the second leading cause of cancer death in women. One in eight American women (13%) will be diagnosed with breast cancer in their lifetime, and one in 39 women (3%) will die from breast cancer (American Cancer Society Statistics, 2023). The American Cancer Society recommends yearly screening mammography for early detection of breast cancer for women, which may begin at age 40 [1]. About 2%–5% of women in the general population in the US have a lifetime risk of breast cancer of 20% or higher [1], although it can vary depending on the population being studied and the risk assessment method used. The ACS recommends yearly breast magnetic resonance imaging (MRI) in addition to mammography for women with 20–25% or greater lifetime risk [1]. Early detection and treatment are likely to result in better patient outcomes.

MRI is generally more sensitive and offers more detailed pathophysiological information but is less cost effective compared to mammography for population-based screening [2, 3]. Breast MRI utilizes high-powered magnets and radio waves to generate 3D images. Cancer yield from MRI alone averages 22 cancers for every 1000 women screened, a rate of cancer detection roughly 10 times that achieved with screening mammography in average-risk women, and roughly twice the yield achieved with screening mammography in high-risk women [4]. Many recent studies have established contrast-enhanced breast MRI as a screening modality for women with a hereditary or familial increased risk for the development of breast cancer [5].

Interpretation of breast cancer on MRI relies on the expertise of radiologists. The growing demand for breast MRI and the shortage of radiologists has resulted in increased workload for radiologists [6, 7], leading to long wait times and delays in diagnosis [8, 9]. Machine learning methods show promise in assisting radiologists, in improving accuracy with the interpretation of breast MRI images and supporting clinical decision-making and improving patient outcomes [10, 11]. By analyzing large datasets of MRIs, machine learning algorithms can learn to identify and classify suspicious areas, potentially reducing the number of false positives and false negatives [11, 12] and thus improving diagnostic accuracy. A few studies have shown that machine learning can outperform radiologists in detecting breast cancer on MRIs [13]. Machine learning could also help to prioritize worklists in a radiology department.

In recent years, deep learning (DL) methods have revolutionized the field of computer vision with wide range of applications, from image classification and object detection to semantic segmentation and medical image analysis [14]. Deep learning is superior to traditional machine learning because of its ability to learn from unstructured or unlabeled data [14]. Unlike traditional machine algorithms which require time-consuming data labeling, deep learning algorithms are more flexible and adaptable as they can learn from data that are not labeled or structured [15]. There have been a few reviews on deep learning breast cancer detection. Oza et al. reviewed detection and classification on mammography [16]. Saba et al. [17] presented a compendium of state-of-the-art techniques for diagnosing breast cancers and other cancers. Hu et al. [18] provided a broad overview on the research and development of artificial intelligence systems for clinical breast cancer image analysis, discussing the clinical role of artificial intelligence in risk assessment, detection, diagnosis, prognosis, and treatment response assessment. Mahoro et al. [10] reviewed the applications of deep learning to breast cancer diagnosis across multiple imaging modalities. Sechopoulos et al. [19] discussed the advances of AI in the realm of mammography and digital tomosynthesis. AI-based workflows integrating multiple datastreams, including breast imaging, can support clinical decision-making and help facilitate personalized medicine [20]. To our knowledge, there is currently no review that systematically compares different deep learning studies of breast cancer detection using MRI. Such a review would be important because it could help to delineate the path forward.

**Fig. 1:**
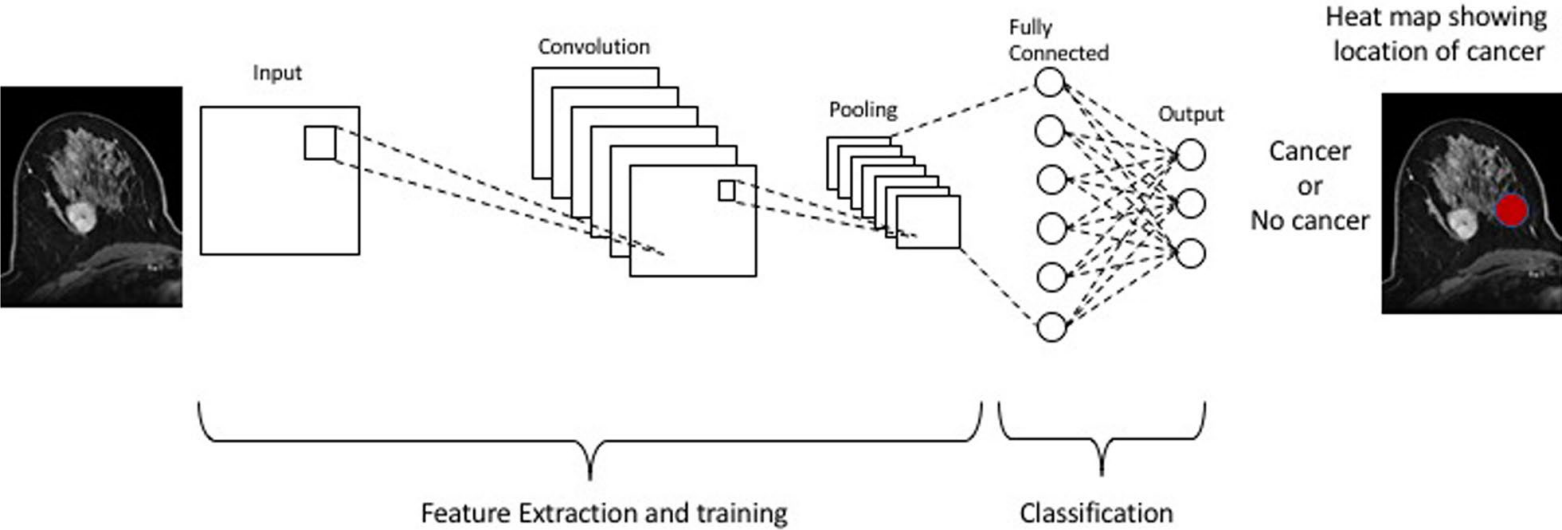
The input layer represents the breast cancer image that serves as input to the CNN. The multiple convolutional layers are stacked on top of the input layer. Pooling layers are used to downsample the spatial dimensions of the feature maps. Fully connected layers are then employed to connect all the neurons from the previous layers to the subsequent layers. The final layer is the output layer, which provides the classification

Figure 1 shows a graphic representation of a deep learning workflow. The input layer represents the breast cancer image that serves as input to the CNN. The multiple convolutional layers are stacked on top of the input layer. Each convolutional layer applies filters or kernels to extract specific features from the input image. These filters learn to detect patterns such as edges, textures, or other relevant features related to breast cancer. After each convolutional layer, activation functions like rectified linear unit (ReLU) are typically applied to introduce nonlinearity into the network. Following some of the convolutional layers, pooling layers are used to downsample the spatial dimensions of the feature maps. Common pooling techniques include max-pooling or average pooling. Pooling helps reduce the computational complexity and extract the most salient features. After the convolutional and pooling layers, fully connected layers are employed. These layers connect all the neurons from the previous layers to the subsequent layers. Fully connected layers enable the network to learn complex relationships between features. The final layer is the output layer, which provides the classification or prediction. In the case of breast cancer detection, it might output the probability or prediction of malignancy or benignity.

The goal of this study was to review the current literature on deep learning detection of breast cancer using breast MRI. We included literature in which DL was used for both primary screening setting and as a supplemental detection tool. We compared different deep learning algorithms, methods of analysis, types of ground truths, sample size, numbers of benign and malignant lesions, MRI image types, and performance indices, among others. We also discussed lessons learned, challenges of deployment in clinical practice and suggested future research directions.

## Materials and methods

No ethics committee approval was required for this review.

### Search strategy and eligibility criteria

**Fig. 2:**
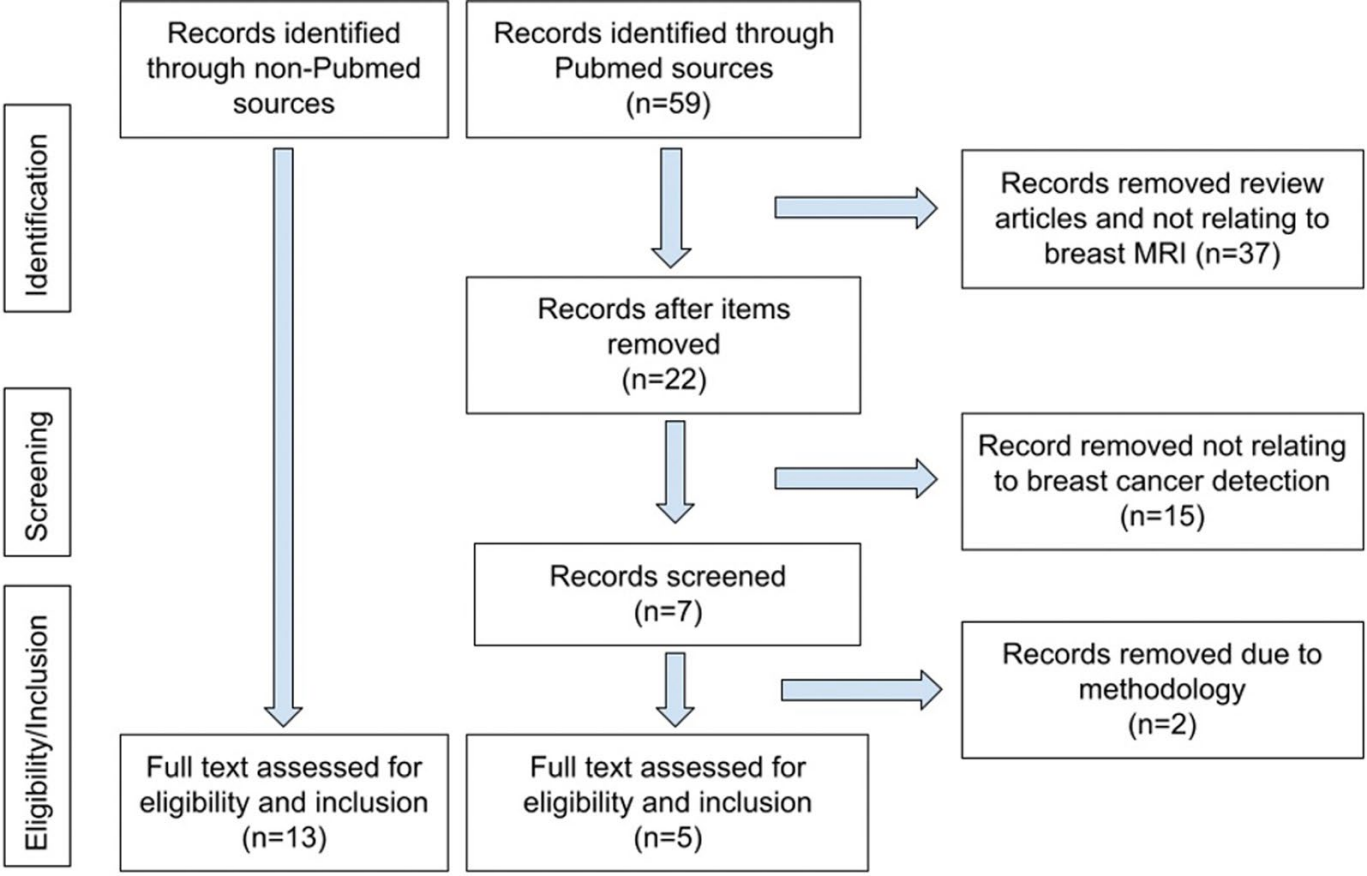
PRISMA selection flowchart

PRISMA guidelines for reporting were adopted in our systematic review. The literature search was performed from 2017 to Dec 31, 2022, using the following key words: “breast MRI,” “breast magnetic resonance imaging,” “deep learning,” “breast cancer detection,” and “breast cancer screening.” The database included Pubmed, Semantic Scholar, ACM Digital Library, Google search, Google Scholar, and pre-print depositories (such as Research Square). We noted that many of the computing or machine learning journals were found on sites other than Pubmed. Some were full-length peer-reviewed conference papers, in contrast with small conference abstracts. Articles that were not deep learning (such as texture analysis) were excluded. Only original articles written in English were selected. Figure 2 shows the flowchart demonstrating how articles were included and excluded for our review. The search and initial screening for eligibility were performed by RA and independently verified by KD and/or TD. This study did not review DL prediction of neoadjuvant chemotherapy which has recently been reviewed [21].

## Results

Pubmed search yielded 59 articles, of which 22 were review articles, 30 were not related to breast cancer detection on MRI, and two had unclear/unconventional methodologies. Five articles were found in Pubmed search after exclusion (Fig. 2). In addition, 13 articles were found on different databases outside of Pubmed, because many computing and machine learning journals were not indexed by Pubmed. A total of 18 articles were included in our study (Table 1). Two of the studies stated that the patient populations were moderate/high risk [22, 23] or high risk [23], while the remaining papers do not state whether the dataset was from screening or supplemental MRI.

**Table 1:** Summary of studies included in this review

Study	Source of data (where data were obtained)	# of patients	Benign lesions	Malignant lesions	Image type	DL architecture	Box or contour	Ground truth	Cross validation	Heatmap	AUC	Accu	Sens	Spec
Adachi [13]	Tokyo Medical and Dental University (Japan)	72	20	52	MIP, DCE	RetinaNet	Bounding box	Rad per path; path, > 1y F/U	Hold-out method	No	0.93	Not reported	0.93	0.83
Antropova [24]	University of Chicago	690	212	478	MIP DCE; DCE subtraction	ConvNetVGGN	Manual ROI with bounding box	Rad per path; biopsy proven	Fivefold	No	0.88, 0.80, 0.83	Not reported	Not reported	Not reported
Ayatollahi [22]	Radbound University Medical Center (the Netherlands)	462	207	365	DCE	RetinaNet	Bounding box	Path, 2y F/U	Tenfold	No	0.90	Not reported	0.95	Not reported
Feng [24]	Tangdu Hospital and Xi'an International Medical Center Hospital (China)	100	32	68	DCE + DWI	KFLI	Bounding box	Path	Hold-out method	No	0.86	0.85	0.85	0.86
Fujioka [25]	Tokyo Medical and Dental University (Japan)	72	12 normal 13 benign	47	MIP DCE	InceptionResNetV2 Others	None	Path, > 1y F/U	Tenfold	Yes	0.83	Not reported	0.75	0.96
Haarburger [26]	University Hospital Aachen (Germany)	408	103	305	DCE + T2	Mask-R-CNN; Retina U-Net; 3D ResNet18 Naive; ResNet18; Curriculum	Coarse localization by radiologist Whole breast images	Path, two-year F/U	Fivefold	Yes	0.88 0.89 0.50 0.89 0.93	0.77 0.82 0.45 0.81 0.93	Not reported	Not reported
Herent [27]	Journées Francophones de Radiologie (France)	335	212	123	Post-contrast T1	CNN Resnet-50	Manual bounding boxes	Path	Threefold	No	0.82	Not reported	Not reported	Not reported
Hu [28]	University of Chicago	616	199	728	DCE, T2 (not coupled)	CNN		Path, Rad reports	Fivefold		0.87	Not reported	0.78	0.79
Li [29]	Zhiejiang Cancer Hospital, China	143	66	77	DCE	2D vs. 3D CNN	Bounding box	Path verified		No	0.80 3D	0.78 3D	0.74 3D	0.82 3D
Liu [30]	Multiple institutions in the US (ISPY-1 data)*	438			DCE	CNN	Square cropping in sagittal plane	Path Rad annotation	Fivefold	No	0.92	0.94	0.74	0.95
Marrone [31]	University of Naples (Italy)	42	25	42	DCE 4D	AlexNet CNN	Manual ROI	Path	Tenfold	No	0.76	0.76	0.83	0.79
Rasti [32]	Imaging Center of Milad Hospital (Tehran)	112	59	53	DCE 1^st^ Post-subtraction	ME-CNN		Path	Fivefold	No	0.99	0.96	0.98	0.95
Truhn [33]	University of Aachen (Germany)	447	507	787	T2, pre- and post-contrast	CNN ResNet 18	Manual segmentation	Path, F/U	Tenfold	No	0.88	Not reported	0.78	0.85
Wu [34]	Beijing University People's Hospital (China)	130	59	71	DCE	CNN	Bounding box	Path		Yes	0.91	0.88	0.86	Not reported
Yurttakal [35]	Haseki Training and Research Hospital (Turkey)	200	98	102	DCE subtraction	CNN	Cropping tumorous regions Rectang box	Path, Rad		No	Not reported	0.98	1.0	0.97
Zheng [36]	Renji Hospital (China)	72	45	27	DCE + DWI Multi-timepoints	DC-LSTM, ResNet50	Labels by radiologists, then cropped 40 × 40x40	Rad per path; Path for B/M benign vs. mal	Threefold	No	Not reported	0.85	Not reported	Not reported
Zhou [37]	*Not specified* (Likely China)	133	62	91	DCE	CNN ResNet50	Segment by fuzzy C-means after radiologist indicated location	Path	Tenfold	No	0.97–0.99	0.89	0.94	0.81
Zhou [38]	The Fifth Medical Center of Chinese PLA General Hospital (China)	307	101	206	DCE	3D DenseNet GAP 3D DenseNet GMP Ensemble	Bounding box	Path, 3y F/U		Yes	0.86 0.86 0.86	0.81 0.81 0.83	0.86 0.92 0.91	0.70 0.61 0.69

Studies are arranged alphabetically. DCE: dynamic contrast enhancement, DWI: diffusion-weighted imaging, MIP: maximum intensity projection, T2: T2-weighted MRI. F/U: follow-up, Rad: radiology, Path: pathology, CNN: convolutional neural network, AUC: area under the curve, Accu: accuracy, Sens: sensitivity, and Spec: specificity

^*^public dataset (see text for link)

In this review, we first summarized individual papers and followed by generalization of lessons learned. We then discussed challenges of deployment in the clinics and suggested future research directions.

### Summary of individual papers

Adachi et al. [13] performed a retrospective study using RetinaNet as a CNN architecture to analyze and detect breast cancer in MIPs of DCE fat-suppressed MRI images. Images of breast lesions were annotated with a rectangular region-of-interest (ROI) and labeled as “benign” or “malignant” by an experienced breast radiologist. The AUCs, sensitivities, and specificities of four readers were also evaluated as well as those of readers combined with CNN. RetinaNet alone had a higher area under the curve (AUC) and sensitivity (0.925 and 0.926, respectively) than any of the readers. In two cases, the AI system misdiagnosed normal breast as malignancy, which may be the result of variations in normal breast tissue. Invasive ductal carcinoma near the axilla was missed by AI, possibly due to confusion for normal axillary lymph node. Wider variety of data and larger datasets for training could alleviate these problems.

Antropova et al. [24] compared MIP derived from the second post-contrast subtraction T1-weighted image to the central slice of the second post-contrast image with and without subtraction. The ground truth was ROIs based on radiology assessment with biopsy-proven malignancy. MIP images showed the highest AUC. Feature extraction and classifier training for each slice for DCE-MRI sequences, with slices in the hundreds, would have been computationally expensive at the time. MIP images, in widespread use clinically, contain enhancement information throughout the tumor volume. MIP images, which represent a volume data, avoid using a plethora of slices, and are, therefore, faster and computationally less intensive and less expensive. MIP (AUC = 0.88) outperformed one-slice DCE image, and subtracted DCE image (AUC = 0.83) outperformed single-slice DCE image (AUC = 0.80). The subtracted DCE image is derived from 2 timepoints, the pre-contrast image subtracted from the post-contrast image, which produces a higher AUC. Using multiple slices and/or multiple timepoints could further increase the AUC with DCE images, possibly even exceeding that of the MIP image (0.88). This would be an area for further exploration.

Ayatollahi et al. [22] performed a retrospective study using 3D RetinaNet as a CNN architecture to analyze and detect breast cancer in ultrafast TWIST DCE-MRI images. They used 572 images (365 malignant and 207 benign) taken from 462 patients. Bounding boxes drawn around the lesion in the images were used as ground truth. They found a detection rate of 0.90 and a sensitivity of 0.95 with tenfold cross validation.

Feng et al. [23] implemented the Knowledge-Driven Feature Learning and Integration model (KFLI) using DWI and DCE-MRI data from 100 high-risk female patients with 32 benign and 68 malignant lesions, segmented by two experienced radiologists. They reported 0.85 accuracy. The model formulated a sequence division module and adaptive weighting module. The sequence division module based on lesion characteristics is proposed for feature learning, and the adaptive weighting module proposed is used for automatic feature integration while improving the performance of cooperative diagnosis. This model provides the contribution of sub-sequences and guides the radiologists to focus on characteristic-related sequences with high contribution to lesion diagnosis. This can save time for the radiologists and helps them to better understand the output results of the deep networks. As such, it can extract sufficient and effective features from each sub-sequence for a comprehensive diagnosis of breast cancer. This model is a deep network and domain knowledge ensemble that achieved high sensitivity, specificity, and accuracy.

Fujioka et al. [25] used 3D MIP projection from early phase (1–2 min) of dynamic contrast-enhanced axial fat-suppressed DCE mages, with performance of CNN models compared to two human readers (Reader 1 = breast surgeon with 5 years of experience and Reader 2 = radiologist with 20 years of experience) in distinguishing benign from malignant lesions. The highest AUC achieved with deep learning was with InceptionResNetV2 CNN model, at 0.895. Mean AUC across the different CNN models was 0.830, and range was 0.750–0.895, performing comparably to human readers. False-positive masses tended to be relatively large with fast pattern of strong enhancement, and false-negative masses tended to be relatively small with medium to slow pattern of enhancement. One false positive and one false negative for non-mass enhancing lesion that was observed were also incorrectly diagnosed by the human readers. The main limitation of their study was small sample size.

Haarburger et al. [26] performed an analysis of 3D whole-volume images on a larger cohort (*N* = 408 patients), yielding an AUC of up to 0.89 and accuracy of 0.81, further establishing the feasibility of using 3D DCE whole images. Their method involved feeding DCE images from 5 timepoints (before contrast and 4 times post-contrast) and T2-weighted images to the algorithms. The multicurriculum ensemble consisted of a 3D CNN that generates feature maps and a classification component that performs classification based on the aggregated feature maps made by the previous components. AUC range of 0.50–0.89 was produced depending on the CNN models used. Multiscale curriculum training improved simple 3D ResNet18 from an AUC of 0.50 to an AUC of 0.89 (ResNet18 curriculum). A radiologist with 2 years of experience demonstrated AUC of 0.93 and accuracy of 0.93. An advantage of the multicurriculum ensemble is the elimination of the need for pixelwise segmentation for individual lesions, as only coarse localization coordinates for Stage 1 training (performed in 3D in this case) and one global label per breast for Stage 2 training is needed, where Stage 2 involved predictions of whole images in 3D in this study. The high performance of this model can be attributed to the high amount of context and global information provided. Their 3D data use whole breast volumes without time-consuming and cost prohibitive lesion segmentation. A major drawback of 3D images is the requirement of more RAM and many patients required to train the model.

Herent et al. [27] used T1-weighted fat-suppressed post-contrast MRI in a CNN model that detected and then characterized lesions (*N* = 335). Lesion characterization consisted of diagnosing malignancy and lesion classification. Their model, therefore, performed three tasks and thereby was a multitask technique, which limits overfitting. ResNET50 neural network performed feature extraction from images, and images were processed by the algorithm’s attention block which learned to detect abnormalities. Images were fed into a second branch where features were averaged over the selected regions, then fitted to a logistic regression to produce the output. On an independent test set of 168 images, a weighted mean AUC of 0.816 was achieved. The training dataset consisted of 17 different histopathologies, of which most were represented as very small percentages of the whole dataset of 335. Several of the listed lesion types represented less than 1% of the training dataset. This leads to the problem of overfitting. The authors mention that validation of the algorithm by applying it to 3D images in an independent dataset, rather than using the single 2D images as they did, would show if the model is generalizable. The authors state that training on larger databases and with multiparametric MRI would likely increase accuracy. This study shows good performance of a supervised attention model with deep learning for breast MRI.

Hu et al. [28] used multiparametric MR images (DCE-MRI sequence and a T2-weighted MRI sequence) in a CNN model including 616 patients with 927 unique breast lesions, 728 of which were malignant. A pre-trained CNN extracted features from both DCE and T2w sequences depicting lesions that were classified as benign or malignant by support vector machine classifiers. Sequences were integrated at different levels using image fusion, feature fusion, and classifier fusion. Feature fusion from multiparametric sequences outperformed DCE-MRI alone. The feature fusion model had an AUC of 0.87, sensitivity of 0.78, and specificity of 0.79. CNN models that used separate T2w and DCE images into combined RBG images or aggregates of the probability of malignancy output from DCE and T2w classifiers both did not perform significantly better than the CNN model using DCE-alone. Although other studies have demonstrated that single-sequence MRI is sufficient for high CNN performance, this study demonstrates that multiparametric MRI (as fusion of features from DCE-MRI and T2-weighted MRI) offers enough information to outperform single-sequence MRI.

Li et al. [29] used 3D CNNs in DCE-MR images to differentiate between benign and malignant tumors from 143 patients. In 2D and 3D DCE-MRI, a region-of-interest (ROI) and volume-of-interest (VOI) were segmented, and enhancement ratios for each MR series were calculated. The AUC value of 0.801 for the 3D CNN was higher than the value of 0.739 for 2D CNN. Furthermore, the 3D CNN achieved higher accuracy, sensitivity, and specificity values of 0.781, 0.744, and 0.823, respectively. The DCE-MRI enhancement maps had higher accuracy by using more information to diagnose breast cancer. The high values demonstrate that 3D CNN in breast cancer MR imaging can be used for the detection of breast cancer and reduce manual feature extraction.

Liu et al. [30] used CNN to analyze and detect breast cancer on T1 DCE-MRI images from 438 patients, 131 from I-SPY clinical trials and 307 from Columbia University. Segmentation was performed through an automated process involving fuzzy C-method after seed points were manually indicated. This study included analysis of commonly excluded image features such as background parenchymal enhancement, slice images of breast MRI, and axilla/axillary lymph node involvement. The methods also minimized annotations done at pixel level, to maximize automation of visual interpretation. These objectives increased efficiency, decreased subjective bias, and allowed for complete evaluation of the whole image. Obtaining images with multiple timepoints from multiple institutions made the algorithm more generalizable. The CNN model achieved AUC of 0.92, accuracy of 0.94, sensitivity of 0.74, and specificity of 0.95.

Marrone et al. [31] used CNN to evaluate 42 malignant and 25 benign lesions in 42 women. ROIs were obtained by an experienced radiologist, and manual segmentation was performed. Accuracy of up to 0.76 was achieved. AUC as high as 0.76 was seen on pre-trained AlexNet versus 0.73 on fine-tuning of pre-trained AlexNet where the last trained layers were replaced by untrained layers. The latter method could allow reduced number of training images needed. The training from scratch AlexNet model is accomplished when AlexNet pre-trained on the ImageNet database is used to extract a feature vector from the last internal CNN layer, and a new supervised training is employed, which yielded the lowest AUC of 0.68 and accuracy of 0.55.

Rasti et al. [32] analyzed DCE-MRI subtraction images from MRI studies (*N* = 112) using a multi-ensemble CNN (ME-CNN) functioning as a CAD system to distinguish benign from malignant masses, producing 0.96 accuracy with their method. The ME-CNN is a modular and image-based ensemble, which can stochastically partition the high-dimensional image space through simultaneous and competitive learning of its modules. It also has the advantages of fast execution time in both training and testing and a compact structure with a small number of free parameters. Among several promising directions, one could extend the ME-CNN approach to the pre-processing stage, by combining ME-CNN with recent advances in fully autonomous CNNs for semantic segmentation.

Truhn et al. [33] used T2-weighted images with one pre-contrast and four post-contrast DCE images in 447 patients with 1294 enhancing lesions (787 malignant and 507 benign) manually segmented by a breast radiologist. Deep learning with CNN demonstrated an AUC of 0.88 which was inferior to prospective interpretation by one of the three breast radiologists (7–25 years of experience) reading cases in equal proportion (0.98). When only half of the dataset was used for training (*n* = 647), the AUC was 0.83. The authors speculate that with increased training on a greater number of cases that their model could improve its performance.

Wu et al. [34] trained a CNN model to analyze and detect lesions from DCE T1-weighted images from 130 patients, 71 of which had malignant lesions and 59 had benign tumors. Fuzzy C-means clustering-based algorithm automatically segmented 3D tumor volumes from DCE images after rectangular region-of-interest were placed by an expert radiologist. An objective of the study was to demonstrate that single-sequence MRI at multiple timepoints provides sufficient information for CNN models to accurately classify lesions.

Yurtakkal et al. [35] utilized DCE images of 98 benign and 102 malignant lesions, producing 0.98 accuracy, 1.00 sensitivity, and 0.96 specificity. The multi-layer CNN architecture utilized consisted of six groups of convolutional, batch normalization, rectified linear activation function layers, and five max-pooling followed by one dropout layer, one fully connected layer, and one softmax layer.

Zheng et al. [36] used a dense convolutional long short-term memory (DC-LSTM) on a dataset of lesions obtained through a university hospital (*N* = 72). The method was inspired by DenseNet and built on convolutional LSTM. It first uses a three-layer convolutional LSTM to encode DCE-MRI as sequential data and extract time-intensity information then adds a simplified dense block to reduce the amount of information being processed and improve feature reuse. This lowered the variance and improved accuracy in the results. Compared to a ResNet-50 model trained only on the main task, the combined model of DC-LSTM + ResNet improved the accuracy from 0.625 to 0.847 on the same dataset. Additionally, the authors proposed a latent attributes method to efficiently use the information in diagnostic reports and accelerate the convergence of the network.

Jiejie Zhou et al. [37] evaluated 133 lesions (91 malignant and 62 benign) using ResNET50, which is similar to ResNET18 used by Truhn et al. [33] and Haarburger et al^.^ [26]. Their investigation demonstrated that deep learning produced higher accuracy compared to ROI-based and radiomics-based models in distinguishing between benign and malignant lesions. They compared the metrics resulting from using five different bounding boxes. They found that using the tumor alone and smaller bounding boxes yielded the highest AUC of 0.97–0.99. They also found that the inclusion of a small amount of peritumoral tissue improved accuracy compared to smaller boxes that did not include peritumoral tissue (tumor alone boxes) or larger input boxes (that include tissue more remote from peritumoral tissue), with accuracy of 0.91 in the testing dataset. The tumor microenvironment influences tumor growth, and the tumor itself can alter its microenvironment to become more supportive of tumor growth. Therefore, the immediate peritumoral tissue, which would include the tumor microenvironment, was important in guiding the CNN to accurately differentiate between benign and malignant tumors. This dynamic peritumoral ecosystem can be influenced by the tumor directing heterogeneous cells to aggregate and promote angiogenesis, chronic inflammation, tumor growth, and invasion. Recognizing features displayed by biomarkers of the tumor microenvironment may help to identify and grade the aggressiveness of a lesion. This complex interaction between the tumor and its microenvironment may potentially be a predictor of outcomes as well and should be included in DL models that require segmentation. In DL models using whole images without segmentation of any sort, the peritumoral tissue would already be included, which would preclude the need for precise bounding boxes.

Juan Zhou et al. [38] used 3D deep learning models to classify and localize malignancy from cases (*N* = 1537) of MRIs. The deep 3D densely connected networks were utilized under image-level supervision (weakly supervised). Since 3D weakly supervised approach was not well studied compared to 2D methods, the purpose of this study was to develop a 3D deep learning model that could identify malignant cancer from benign lesions and could localize the cancer. The model configurations of global average pooling (GAP) and global max-pooling (GMP) that were used both achieved over 0.80 accuracy with AUC of 0.856 (GMP) and 0.858 (GAP) which demonstrated the effectiveness of the 3D DenseNet deep learning method in MRI scans to diagnose breast cancer. The model ensemble achieved AUC of 0.859.

### Summary of lessons learned

Most studies were single-center studies, but they came from around the world, with the majority coming from the US, Asia, and Europe. All studies except one [33] were retrospective studies. The sample size of each study ranged from 42 to 690 patients, generally small for DL analysis. Sample sizes for patients with benign and malignant lesions were comparable and were not skewed toward either normal or malignant lesions, suggesting that these datasets were not from high-risk screening patients because high-risk screening dataset would have consisted of very low (i.e., typically < 5%) positive cases.

#### Image types

Most studies used private datasets as their image source. ISPY-1 data were the only public dataset noted (https://wiki.cancerimagingarchive.net/pages/viewpage.action?pageId=20643859). Most studies involved DCE data acquisition, but most analysis include only a single post-contrast MRI. For those that used multiple post-contrast MRI dynamics, most fed each dynamic into each separate independent channel, which does not optimally make use of the relationships between imaging dynamics. Some studies used subtraction of post- and pre-contrast or signal enhancement ratio (SER) [24, 32, 35]. Three studies utilized MIP DCE images to minimize computation cost [13, 24, 25]. However, collapsing images by MIP has drawbacks (i.e., collapse enhanced vascular structures into a single plane may be mistaken as cancer). There were only five studies [23, 26, 28, 33, 36] that utilized multiparametric data types (i.e., DCE, T2-weighted, and DWI). Although combining multiple types of MRIs should improve performance, this has not been conclusively demonstrated in practice.

#### Types of DL architectures

RetinaNet and KFLi are optimized for object detection, while VGGNet, InceptionResNet, and AlexNet are designed for image classification (see review [16, 17, 39]). LSTM is used for time-series modeling. DenseNet, on the other hand, can be used for a wide range of tasks, including image classification, object detection, and semantic segmentation. Ensemble methods, which combine multiple models, are useful for boosting the overall performance of a system. U-Net and R-Net are specialized deep learning models for semantic segmentation tasks in medical image analysis. U-Net uses an encoder–decoder architecture to segment images into multiple classes, while R-Net is a residual network that improves the accuracy and efficiency of the segmentation task.

The most used algorithm is CNN or CNN-based. There is no consensus that certain algorithms are better than others. Given the fact that different algorithms were tested on different datasets, it is not possible to conclude that a particular DL architecture performs better than others. Careful comparison of multiple algorithms on the same datasets is needed. Thus, we only discussed potential advantages and disadvantages of each DL architecture. Performance indices could be misleading.

Although each model has its own unique architecture and design principles, most of the above-mentioned methods utilized convolutional layers, pooling layers, activation functions, and regularization techniques (such as dropout and batch normalization) for model optimization. Additionally, the use of pre-trained models and transfer learning has become increasingly popular, allowing leverage of knowledge learned from large datasets such as ImageNet to improve the performance of their models on smaller, specialized datasets. However, the literature on transfer learning in breast cancer MRI detection is limited. A relatively new deep learning method known as transformer has found exciting applications in medical imaging [40, 41].

#### Ground truths

Ground truths were either based on pathology (i.e., benign versus malignant cancer), radiology reports, radiologist annotation (ROI contoured on images), or a bounding box, with reference to pathology or clinical follow-up (i.e., absence of a positive clinical diagnosis). While the gold standard is pathology, imaging or clinical follow-up without adverse change over a prescribed period has been used as empiric evidence of non-malignancy. This is an acceptable form of ground truth.

#### Heatmaps

Only four out of 18 studies provided heatmaps of the regions that the DL algorithms consider important. Heatmaps enable data to be presented visually in color showing whether the area of activity makes sense anatomically or if it is artifactual (i.e., biopsy clip, motion artifact, or outside of the breast). Heatmaps are important for interpretability and explainability of DL outputs.

#### Performance

All studies include some performance indices, and most include AUC, accuracy, sensitivity, and specificity. AUC ranged from 0.5 to 1.0, with the majority around 0.8–0.9. Other metrics also varied over a wide range. DL training methods varied, and they included leave-one-out method, hold-out method, and splitting the dataset (such as 80%/20% training/testing) with cross validation. Most studies utilized five- or tenfold cross validation for performance evaluation but some used a single hold-out method, and some did not include cross validation. Cross validation is important to avoid unintentional skewing of data due to partition for training and testing. Different training methods could affect performance. Interpretation of these metrics needs to be made with caution as there could be study reporting bias, small sample size, and overfitting, among others. High-performance indices of the DL algorithm performance are necessary for adoption in clinical use. However, good performance indices alone are not sufficient. Other measures such as heatmaps and experience to gain trust are needed for widespread clinical adoption of DL algorithms.

### DL detection of axillary lymph node involvement

Accurate assessment of the axillary lymph node involvement in breast cancer patients is also essential for prognosis and treatment planning [42, 43]. Current radiological staging of nodal metastasis has poor accuracy. DL detection of lymph node involvement is challenging because of their small sizes and difficulty in getting ground truths. Only a few studies have reported the use of DL to detect lymph node involvement [44–46].

### Challenges for DL to achieve routine clinical applications

Although deep learning is a promising tool in the diagnosis of breast cancer, there are several challenges that need to be addressed before routine clinical applications can be broadly realized.

Data availability: One of the major challenges in medical image diagnosis (and breast cancer MRI in particular) is the availability of large, diverse, and well-annotated datasets. Deep learning models require a large amount of high-quality data to learn from, but, in many cases, medical datasets are small and imbalanced. In medical image diagnosis, it is important to have high-quality annotations of images, which can be time-consuming and costly to obtain. Annotating medical images requires specialized expertise, and there may be inconsistencies between different experts. This can lead to challenges in building accurate and generalizable models. Medical image datasets can lack diversity, which can lead to biased models. For example, a model trained on images with inadequate representation of racial or ethnicity subgroups may not be broadly generalizable. Private medical datasets obtained from one institution could be non-representative of certain racial or ethnic subgroups and, therefore, may not be generalizable. Publicly available data are unfortunately limited, one of which can be found on cancerimagingarchive.net. Collaborative learning facilitating training of DL models by sharing data without breaching privacy can be accomplished with federated learning [47].

Interpretability**,** explainability, and generalizability [48]: Deep learning models are often seen as “black boxes” that can be difficult to interpret. This is especially problematic in medical image diagnosis, where it is important to understand why a particular diagnosis is made. Recent research has focused on developing methods to explain the decision-making process of deep learning models, such as using attention mechanisms or generating heatmaps to highlight relevant regions in the MRI image. While efforts have been made to develop methods to explain the decision-making process of deep learning models, the explainability of these models is still limited [49]. This can make it difficult for clinicians to understand the model's decision and to trust the model. Deep learning models may perform well on the datasets on which they were trained but may not generalize well to new datasets or to patients with different characteristics. This can lead to challenges in deploying the model in a real-world setting.

Ethical concerns: Deep learning models can be used to make life-or-death decisions, such as the diagnosis of cancer. This raises ethical concerns about the safety, responsibility, privacy, fairness, and transparency of these models [50]. There are also social implications (including but not limited to equity) of using artificial intelligence in health care. This needs to be addressed as we develop more and more powerful DL algorithms.

## Perspectives and conclusions

Artificial intelligence has the potential to revolutionize breast cancer screening and diagnosis, helping radiologists to be more efficient and more accurate, ultimately leading to better patient outcomes. It can also help to reduce the need for biopsy or unnecessary testing and treatment. However, some challenges exist that preclude broad deployment in clinical practice to date. There need to be large, diverse, and well-annotated images that are readily available for research. Deep learning results need to be more accurate, interpretable, explainable, and generalizable. A future research direction includes incorporation of other clinical data and risk factors into the model, such as age, family history, or genetic mutations, to improve diagnostic accuracy and enable personalized medicine. Another direction is to assess the impact of deep learning on health outcomes to enable more investment in hospital administrators and other stakeholders. Finally, it is important to address the ethical, legal, and social implications of using artificial intelligence.

## Data Availability

Not applicable.
